# Theoretical study of chemical reactivity descriptors of some repurposed drugs for COVID-19

**DOI:** 10.1557/s43580-023-00590-6

**Published:** 2023-05-31

**Authors:** Razieh Morad, Mahmood Akbari, Malik Maaza

**Affiliations:** 1 UNESCO-UNISA-iTLABS Africa Chair in Nanoscience & Nanotechnology (U2ACN2), College of Graduate Studies, University of South Africa (UNISA), Pretoria, South Africa; 2 Material Research Division, Nanoscience African Network (NANOAFNET), iThemba LABS-National Research Foundation, Somerset West, 7129 South Africa

## Abstract

This study focuses on computational studies of chemical reactivity descriptors of some proposed drugs for COVID-19. Density functional theory calculations were used to optimize the structure and investigate the frontier orbitals and the chemical reactivity descriptors of these drugs. The frontier orbitals, which include both the highest occupied molecular orbital (HOMO) and the lowest unoccupied molecular orbital (LUMO), play an essential role in molecular interactions and chemical reactivity of molecule. Polarizability, which determines the response of the susceptibility of a molecule to an approaching charge, is higher in the more complex drugs such as Hydroxychloroquine, Remdesivir, and Ivermectin compare to the smaller drugs. The HOMO and LUMO orbital energies were calculated to obtain the energy gap of the studied drugs, which is in the following order: Favipiravir < Hydroxychloroquine, Remdesivir < Ivermectin < Artesunate < Artemether < Artemisinin. Generally, molecules with a larger energy gap have lower chemical reactivity and higher kinetic stability.

**Graphical abstract:**

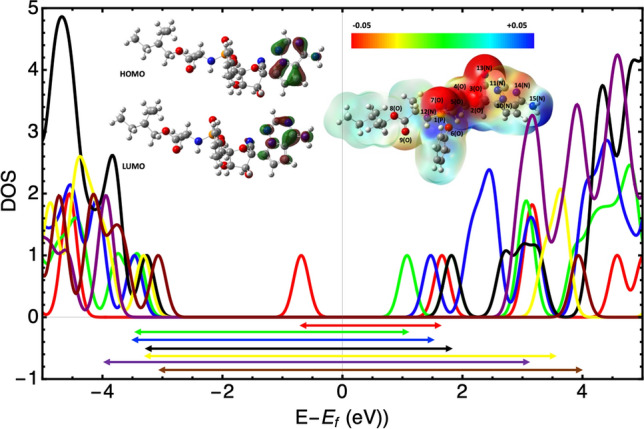

## Introduction

Nanotechnology, a relatively new and rapidly developing discipline involving the formation, management, and application of nano-sized structures [1], has been used in medicine for therapeutic drug delivery and the development of treatments for various diseases and disorders. One of the most important disadvantages of drugs used in therapies of most fatal illnesses like AIDS, cancer, and recently SARS-COV-2 is their non-specific targeting, which demands high dose administration, leading to increased side effects and toxicity [2]. The nano-sized drug delivery systems such as nanoparticles can change the drug biodistribution, promote specific drug targeting, and control drug-release rate, improving the treatment’s efficacy and safety [3]. Antimicrobial drugs such as Artemisinin, Hydroxychloroquine, Favipiravir, Ivermectin, and Remdesivir, have demonstrated promise efficacy against SARS-CoV-2 in clinical studies for COVID-19. However, additional clinical studies were suggested to ensure the safety and efficacy of these drugs [4, 5]. Therefore, further research is required to optimize safe therapeutic techniques.

Molecular modelling has become a very practical and powerful tool to explain the molecular structure and chemical reactivity. Theoretical calculations can be used to explain the reactivity of some compounds in comparison to others and also describe why some sites of the molecule are more reactive than other sites. This study presents the theoretical calculations and computational methods to obtain an insight into chemical reactivity descriptors of the drugs used in the past months of the COVID-19 pandemic [4, 5]. The highest occupied molecular orbital (HOMO) and the lowest unoccupied molecular orbital (LUMO), referred to as frontier orbitals, play a significant role in chemical reactivity and molecular interactions [6]. Studies show that the Kohn–Sham (KS) orbitals have the same order, symmetry, and shape as orbitals using Hartree–Fock theory and, therefore, can describe the various properties of molecules qualitatively. Generally, molecules with a larger energy gap have lower chemical reactivity and higher kinetic stability.

### Computational details

Structures of drug molecules were optimized by performing density functional theory (DFT) at the level of B3LYP functional and the 6–311 +  + g(d,p) basis set implemented in the Gaussian 09 software [7]. At the same level of theory, frequency calculations were done on the optimized structures, and the total energy, dipole moment, and polarizability were reported. The many characteristics of molecules can be naturally described by their molecular orbitals. Especially, the highest occupied molecular orbital (HOMO) and the lowest unoccupied molecular orbital (LUMO), referred to as frontier orbitals, play a significant role in chemical reactivity and molecular interactions [6]. The DFT theory uses Kohn–Sham orbitals, despite the fact that the concept of an orbital originated from a single configuration, such as the Hartree–Fock (HF) theory. However, Studies show that the KS orbitals have the same order, symmetry, and shape as orbitals using HF theory and, therefore, can describe the various properties of molecules qualitatively [8, 9].

The frontier orbital energies (HOMO and LUMO) were obtained for the optimized structure of each drug. Then, the quantum molecular descriptors were calculated as [8]

$${E}_{gap}= {E}_{LUMO}- {E}_{HOMO}$$

$$\mu = \frac{{E}_{LUMO}+ {E}_{HOMO}}{2}$$

$$\eta = \frac{{E}_{LUMO}- {E}_{HOMO}}{2}$$

$$s= \frac{1}{2} \eta$$

$$\chi = -\frac{{E}_{LUMO}+ {E}_{HOMO}}{2}$$

$$\omega = \frac{{\chi }^{2}}{2 \eta }$$

The chemical potential (μ) can be used to assess the evasion affinity of a molecule from equilibrium. The chemical hardness (η) is a property that quantifies the charge transfer and chemical reactivity of a molecule. The less chemical softness (s) indicates the higher stability of the molecule. Electronegativity (χ) determines the ability of a molecule to attract electrons, and finally higher value of electrophilicity index (ω) means higher electrophilic power of the molecule [8].

## Discussion

**Fig. 1:**
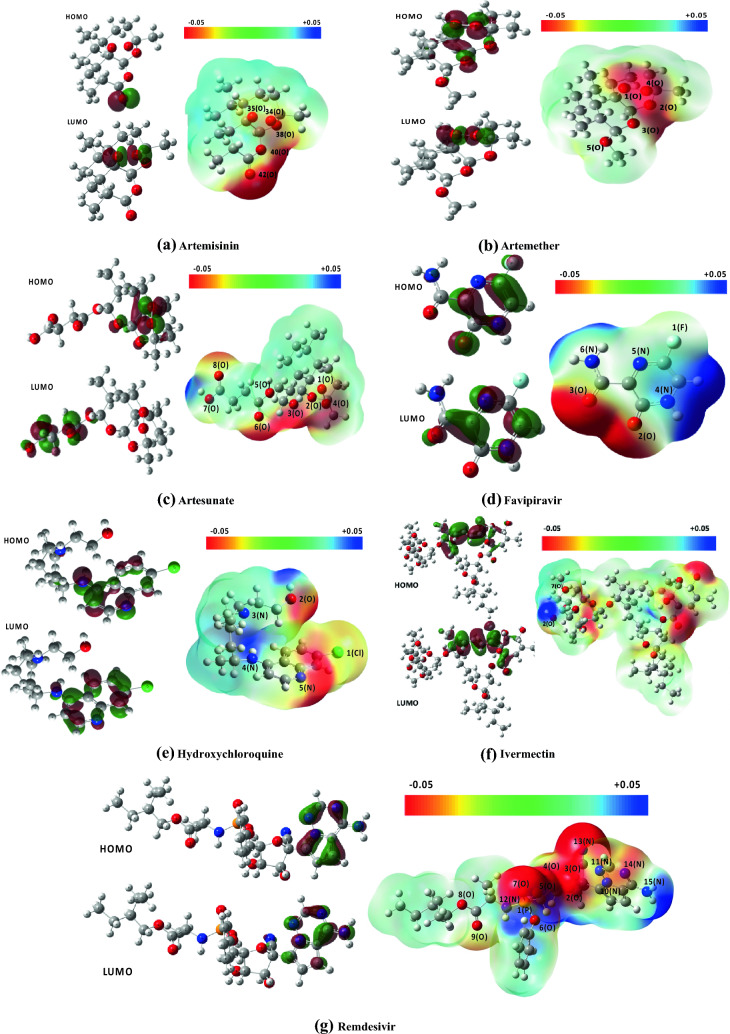
HOMO and LUMO orbitals and molecular electrostatic potential (MEP) map for the studied drugs

The optimized structure of drugs is presented in Fig. 1. All structures are stable, as evidenced by the lack of the imaginary frequency. The results of total energy, total enthalpy, total Gibbs free energy, dipole moment, and the polarizability of the drugs are summarized in Table 1. The high dipole moments could represent their binding position within a specific target protein. The polarizability, which depends on the complexity and size of the molecule, determines how the susceptibility of molecule cloud be affected by approaching a charge. More complex drug molecules such as Hydroxychloroquine, Remdesivir, and Ivermectin have large polarizability, while the Favipiravir, the smallest drug studied here, has the least polarizability.

**Table 1:** Total energy (Hartree), Dipole moment (Debye), and polarizability (a.u) for the optimized structures at the B3LYP/6–311 +  + g(d,p) level of theory

Drug	Total energy	Total enthalpy	Total gibbs free	Dipole moment	Polarizability
Artemisinin	− 960.794175	− 960.775757	− 960.837120	8.533717	245.627667
Artemether	− 1001.007391	− 1000.986836	− 1001.052917	2.933171	266.356736
Artesunate	− 1342.199950	− 1342.174526	− 1342.254246	4.623168	318.711
Favipiravir	− 607.387389	− 607.377134	− 607.422983	8.069670	121.905189
Hydroxychloroquine	− 1400.860133	− 1400.835250	− 1400.916813	11.001991	363.975601
Ivermectin	− 2925.588214	− 2925.522151	− 2925.695329	7.882634	723.397927
Remdesivir	− 2321.056626	− 2321.017912	− 2321.131051	12.547405	543.515664

**Fig. 2:**
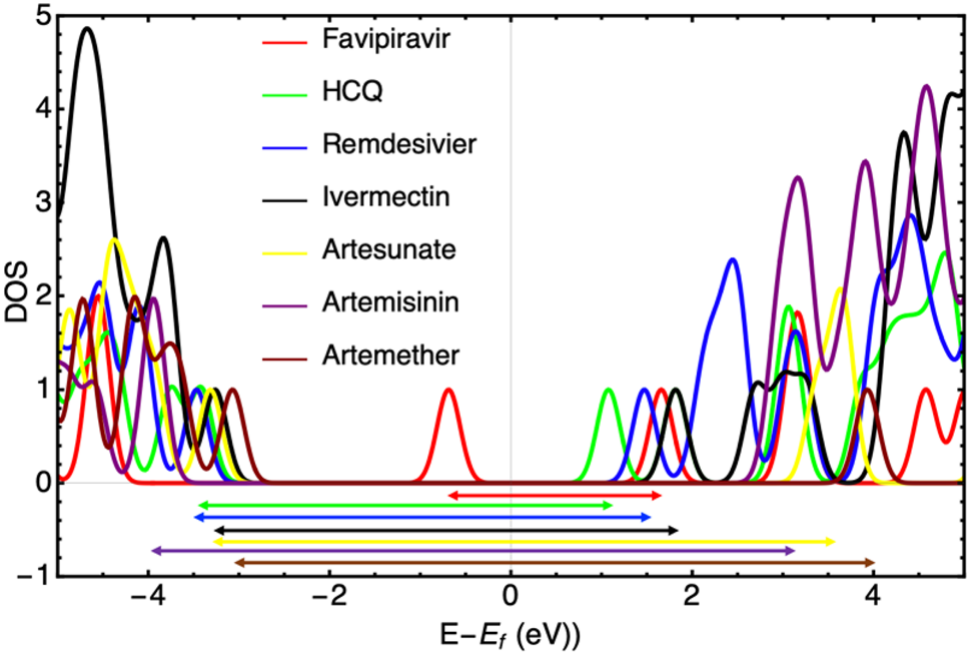
Total density of states of drug molecules. All date shifted in terms of individual molecule Fermi energies such that zero indicates the Fermi energy of each molecule

The HOMO and LUMO energies and the values of chemical reactivity descriptors are reported in Table 2. The HOMO and LUMO orbitals of the studied drugs are shown in Fig. 1. Our DFT results indicate that the HOMO energy of Artemisia drugs is lower than the other drugs, while they have the largest gap energy. The energy gap of the studied drugs is in the following order: Favipiravir < HCQ, Remdesivir < Ivermectin < Artesunate < Artemether < Artemisinin. Generally, soft molecules with a small energy gap are more reactive than harder molecules because they can quickly transfer electrons to acceptors. The density of states (DOS) of all molecules were presented in Fig. 2. The arrows in this figure indicates the band gap of each molecule (color coded) for a better visual comparison.

**Table 2:** The HOMO and LUMO energy, energy gap (eV), chemical potential$$(\mu )$$, chemical hardness ($$\eta )$$,$$)$$, chemical softness ($$s)$$, electronegativity $$\left(\chi \right),$$ and electrophilicity $$\left(\omega \right),$$ (in eV) of investigated drugs in a water solvent

Drugs	${E}_{HOMO}$	${E}_{LUMO}$	${E}_{gap}$	$\mu$	$\eta$	$s$	$\chi$	$\omega$
Artemisinin	− 7.47	− 0.59	6.88	− 4.03	3.44	0.30	4.03	2.36
Artemether	− 7.07	− 0.33	6.73	− 3.70	3.37	0.30	3.70	2.04
Artesunate	− 7.19	− 0.51	6.68	− 3.85	3.34	0.30	3.85	2.22
Favipiravir	− 6.89	− 2.98	3.91	− 4.93	1.95	0.51	4.93	6.23
Hydroxychloroquine	− 5.97	− 1.67	4.30	− 3.82	2.15	0.46	3.82	3.40
Ivermectin	− 5.92	− 0.83	5.08	− 3.37	2.54	0.39	3.37	2.24
Remdesivir	− 6.41	− 1.55	4.80	− 3.98	2.43	0.41	3.98	3.25

The molecular electrostatic potential (MEP) map is a helpful illustration of the reactivity of the drug. MEP visualizes the electronic density in the molecules’ sites such that the red region, which indicates the negative regions of electrostatic potential with the high electron density, are the most active sites. The MEP map of studied drugs calculated by the same method under the same basis sets is illustrated in Fig. 1. For the sake of better comparison, the color scale for all MEPs is fixed.

For Artemisinin, oxygen atom (number 42) with − 0.33e of Hirshfeld charge is the most active site, as shown in Fig. 1a has the highest contribution on HOMO orbital. Oxygen atoms numbers 38, 40, 34, and 35 have the next highest negative Hirshfeld charge (− 0.17, − 0.16, − 0.11, − 0.10). In the Artemether molecule (see Fig. 1b), the four oxygen atoms (atom numbers 1–4) with the highest negative charge are the most active sites and have the main contribution to the HOMO orbital. In the case of Artesunate (see Fig. 1c), the most active sites are the atoms number 8 and 6 (O) with − 0.3e of Hirshfeld charge. Two oxygen atoms with a significant negative electronic potential and Hirshfeld charge of − 0.35e are the most active sites of the Favipiravir molecule (see Fig. 1d). The oxygen atom (atom number 2) and nitrogen number 5 (see Fig. 1e). are the most active sites of HCQ with − 0.26e and − 0.22e of Hirshfeld charge, respectively. Ivermectin molecule has 13 oxygen atoms with a negative Hirshfeld charge of the order of − 0.2e, but the most active sites in this large molecule are the oxygen atoms number 101 and 113 with − 0.28 and − 0.25 Hirshfeld charge, respectively (see Fig. 1f). Oxygen number 7 is the most active site of the Remdesivir drug with the large negative Hirshfeld charge − 0.43e (see Fig. 1g).

## Conclusions

Density functional theory calculations were used to optimize the structure and investigate the frontier orbitals and the chemical reactivity descriptors of these drugs. The frontier orbitals, which include both the highest occupied molecular orbital (HOMO) and the lowest unoccupied molecular orbital (LUMO), play an essential role in molecular interactions and chemical reactivity of molecule [55]. Polarizability, which determines the response of the susceptibility of a molecule to an approaching charge, is higher in the more complex drugs such as Hydroxychloroquine, Remdesivir, and Ivermectin compare to the smaller drugs. The HOMO and LUMO orbital energies were calculated to obtain the energy gap of the studied drugs, which is in the following order: Favipiravir < Hydroxychloroquine, Remdesivir < Ivermectin < Artesunate < Artemether < Artemisinin. Generally, molecules with a larger energy gap have lower chemical reactivity and higher kinetic stability.
